# The Distribution of Cytotoxic Necrotizing Factors (CNF-1, CNF-2, CNF-3) and Cytolethal Distending Toxins (CDT-1, CDT-2, CDT-3, CDT-4) in *Escherichia coli* Isolates Isolated from Extraintestinal Infections and the Determination of their Phylogenetic Relationship by PFGE

**DOI:** 10.1155/2022/7200635

**Published:** 2022-11-16

**Authors:** Cansu Onlen Guneri, Fatih Koksal, Suna Kizilyildirim, Basak Bedir, Togrul Nagiyev

**Affiliations:** ^1^Department of Medical Laboratory Techniques, Medical Microbiology, Gulhane Vocational School of Health Services, University of Health Sciences, 06018 Ankara, Turkey; ^2^Department of Medical Microbiology, Medical Faculty, Cukurova University, 01380 Adana, Turkey; ^3^Department of Pharmaceutical Microbiology, Faculty of Pharmacy, Suleyman Demirel University, 32260 Isparta, Turkey

## Abstract

**Backgrounds:**

Diagnostic markers of extraintestinal infection in *Escherichia coli* (*E. coli*) remain unclear in the literature. Extraintestinal pathogenic *E. coli* (ExPEC) is differentiated from other *E. coli* isolates in terms of virulence factors, such as host cell adhesion, invasion, cytotoxic necrotizing factor (*CNF* (*cnf1-cnf3*)) and cytolethal distending toxin (*CDT* (*cdt1-cdt4*) that are responsible for cell death. We aimed to investigate the frequency of *CNF-CDT* and the relationship between the clinical diagnosis and genotypes in *E. coli* isolates with different clinical origins.

**Methods:**

A total of 646 *E. coli* isolates (obtained from 645 patients) isolated from different infection sites other than the intestine were evaluated in aspects of the *CNF*, *CDT* virulence genes, phylogenetic grouping, and phylogenetic relationship by using PCR and PFGE.

**Results:**

At least one virulence gene was present in 156 (24%) of the 646 ExPEC isolates. We detected *cnf1*, *cnf2*, and *cnf3*, in 78, 12, and 20 ExPEC isolates, respectively. Also, *cdt1*, *cdt2*, *cdt3*, and *cdt4* genes were present in 20, 4, 4, and 4 isolates, respectively. Some isolates harbored more than one gene, being *cnf1*-*cnf3* (*n* = 6), *cnf1*-*cdt1* (*n* = 4), and *cdt1*-*cdt4* (*n* = 4). These 156 isolates were distributed into 106 large clusters by PFGE. Virulent ExPEC is primarily related to groups *B*2 (60%) and *D* (32%).

**Conclusion:**

To our knowledge, this study demonstrated the presence of *cnf2, cnf3, cdt1, cdt2, cdt3*, and *cdt4* genes for the first time in the literature for Turkey. The widespread presence of the CNF gene in *E. coli* helps distinguish ExPEC from commensal isolates.

## 1. Introduction

*Escherichia coli* (*E. coli*) is one of the important members of the human colon microflora. *E. coli* can easily transfer virulence and resistance genes from other bacteria in the intestinal flora. Some genotypes and serotypes of *E. coli* may cause pathologies in the intestinal tract, ranging from mild secretory diarrhoea to inflammatory mortal dysenteric diarrhoea. However, their colonization in sterile areas of the body, outside of the intestinal system, is always considered to be the cause of clinical pathology. Extraintestinal pathogenic *E. coli* (ExPEC) causes urinary tract infections, newborn meningitis, sepsis, osteomyelitis, pneumonia, surgical site infections, and infections in other extraintestinal areas. Although infections caused by ExPEC have low morbidity, it is becoming an increasingly important endemic problem due to its fatal course and prolonged hospital stay [1, 2].

Important virulence mechanisms that distinguish ExPEC from other *E. coli* isolates include the ability to adhere, invasion into host cells/tissues, defects in host defense, and secreted effectors that affect host cellular functions directly. These effectors are composed of a functional toxin and damage the cell cycle, which is the main process of the host cell. Two important toxins/factors have been defined in *E. coli* species: the first one is cytotoxic necrotizing factor (*CNF*), consisting of *cnf1*-*cnf3*, which is a Rho-GTPase-targeting toxin, and the second one is cytolethal distending toxin (*CDT*), consisting of *cdt1*-*cdt4,* which is a genotoxin that may cause DNA damage in target cells. While *CNF*s promote DNA replication without cytokinesis, *CDT*s block mitosis [3, 4]. These toxins-factors are encoded by mobile elements (e.g., the island of pathogenicity, plasmids) [5].

ExPEC-related infections, which have a wide range of disease spectrum from urinary tract infections to fatal bacteraemia, have increased from 17.8% to 65.3% in Turkey in the last 15 years [6]. This increase may be due to ExPEC isolates, which have undergone the pressure of irrational antibiotic use and gained new virulent characteristics with variable genetic elements from different bacteria to survive. There are a limited number of clinical-epidemiological studies showing that ExPEC isolates have different colonization and virulence factors that affect the prognosis of the diseases compared with intestinal *E*. *coli*. These virulence factors are encoded by gene polymorphism.

We designed this study because of the rapid increase in ExPEC-related infections in recent years, the irrational use of antibiotics, and the limited number of studies on this topic in Turkey. We aimed to investigate the frequency of *CNF*-*CDT* and the relationship between the clinical picture and genotypes in *E. coli* isolates with different clinical origins. Thus, it was to detect a specific marker for diagnosing noncommensal *E. coli*.

## 2. Materials and Methods

### 2.1. Collection, Isolation, and Identification of the Specimens

A total of 646 clinical samples obtained from 645 patients were sent from different departments to the Microbiology Laboratories of Çukurova University and Adana Regional Hospitals, Turkey, between September 2014 and April 2016. These 646 ExPEC isolates were isolated from 465 outpatients and 180 inpatients. Also, 80% of all specimens were from the urinary tract, and most of 645 of them were from the urology and paediatric departments. The remaining specimens were collected from other medical departments and belonged to wounds, blood, sputum, tracheal aspirate, sterile body fluid specimens (cerebral spinal fluid and bone marrow), and vaginal swabs. All isolates of *E. coli* isolated from different infection sites other than the intestine during the study period were included consecutively in the present study. In addition, from one patient with the diagnosis of urosepsis, two specimens were obtained; one specimen belonged to blood, and the other one belonged to urine. The median age of patients was 45 years (a range between newborn to 82 years). Urine samples belonged to 80 males and 442 females; other specimens, such as wounds and blood, belonged to 107 males and 16 females. The samples were cultured on blood agar and Endo agar plates (Merck, Germany) at 37°C for 24 hours. Isolates were first evaluated by Gram staining to examine the morphology of colonies and biochemical test characteristics. İsolates were confirmed phenotypically by the IMVIC test.

### 2.2. Storage and General Processes

*E. coli* isolates were kept at −70°C in broth containing 20% glycerol. These isolates were also genotypically confirmed by PCR in the gene region (uidA) encoding the Beta-D-glucuronidase enzyme found structurally in *E. coli* [7]. All isolates of *E. coli* were evaluated in terms of the *CNF* and *CDT* virulence genes, phylogenetic grouping, and phylogenetic relationship. The *uidA* gene and *cdt1*, *cdt2, cdt3*, and *cdt4* genes were amplified in simplex PCRs. However, a multiplex PCR protocol was used to investigate the *cnf1*, *cnf2*, and *cnf3* genes [8, 9]. In addition, the Triplex PCR was used for the phylogenetic grouping of ExPEC isolates [10]. After that, the phylogenetic relationship of ExPEC isolates was investigated by the XbaI-PFGE [11].

### 2.3. PCR Processing

DNA extraction of all isolates was performed mechanically with a Mickle cell disruptor (The Mickle Lab. Engineering Co. Ltd., Gomshall, Surrey, UK). The amplicons were obtained by PCR using previously published primers (Table 1), *uidA*, *cnf1*, *cnf2*, *cnf3*, *cdt1*, *cdt2*, *cdt3*, *cdt4*, and extracted DNA samples.

The PCR amplification was carried out in a total volume of 25 *μ*l. The PCR mixture was constructed as follows: 12.5 *μ*l PCR master mix (1*x* without MgCI2, 4 *μ*l MgCI_2_ (25 mM), 1 *μ*l dNTP (200 *μ*M each nucleotide), 1 *μ*l each primer, Taq DNA polymerase 0.2 *μ*l U (5 U/*μ*l), template DNA 5 *μ*l (approximately 50 ng), and distilled water by completing to 25 microliters.

The *uidA* gene and *cdt1*, *cdt2*, *cdt3*, and *cdt4* genes were amplified in a single PCR reaction in one tube. An in-house PCR method is used for the amplification of *CDT* gene regions.

A multiplex PCR protocol was used to determine the *cnf1*, *cnf2*, and *cnf3* genes. The PCR processes were applied for the amplification of toxin genes (Table 2).

The PCR products were separated on a 2% agarose gel for *cdt*s and *uidA* and a 1.8% agarose gel for *CNF*s and monitored using a Kodak UV transilluminator (Kodak, New York, USA).

Since this was one of the rare studies in Turkey, a positive control isolate for *CNF*-*CDT* genes could not be obtained. After reviewing the literature, positive isolates for amplification were obtained by trying different PCR mixtures and PCR cycles. Then, we used our own isolates as positive controls for the following PCR tests.

### 2.4. Phylogenetic Classification

We investigated the phylogenetic analyses of 156 ExPEC isolates by the Triplex PCR method, which was defined by Clermont previously [10].

*E. coli* isolates belonged to four groups (*A*, *B*1, *B*2, and *D*) based on the presence of the *chuA* and *yjaA* genes and the DNA fragment (TSPE4.C2) (Table 1) by the Triplex PCR (Table 2).

### 2.5. XbaI Pulsed-Field Gel Electrophoresis

PFGE was performed to determine DNA profiles of *CNF*s-*CDT*s producing isolates using the XbaI enzyme as reported previously [11]. This method has been used for *Klebsiella*, *E. coli, P. aeruginosa*, and Acinetobacter (KEPA). Epidemiological relationships between isolates were assessed by studying the PFGE patterns of genomic DNA after restriction by XbaI . XbaI-PFGE is the gold standard for determining the clonal distribution of ExPEC isolates.

## 3. Results

**Figure 1:**
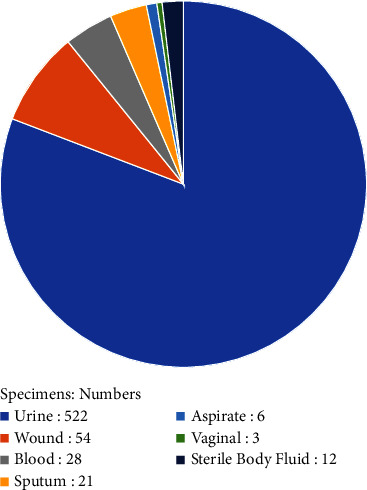
The distribution of isolates by specimens.

**Figure 2:**
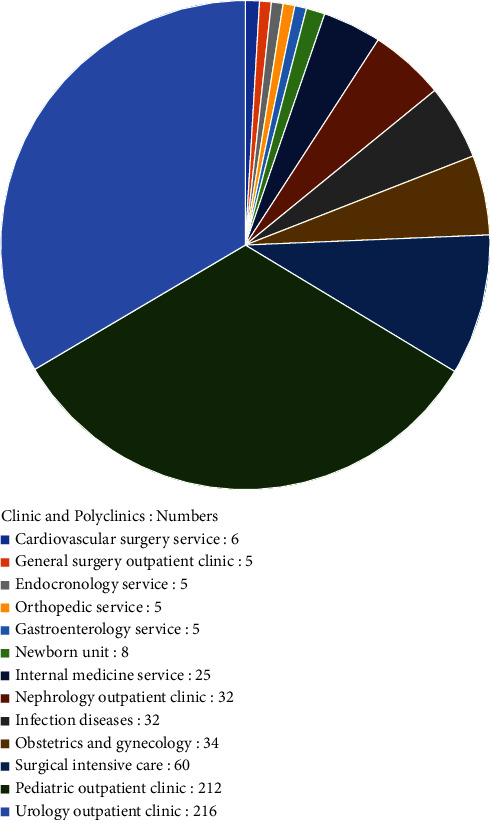
The distribution of 645 patients by clinics and polyclinics.

A total of 646 specimens isolated from the extraintestinal region of the patients within 18 months were included in this study. Of the 646 samples, 522 were urine; of the remaining were 54 wounds, 28 blood, 21 sputum, 6 tracheal aspirates, 12 sterile body fluids, and 3 vaginal swabs (Figures 1 and 2).

**Figure 3:**
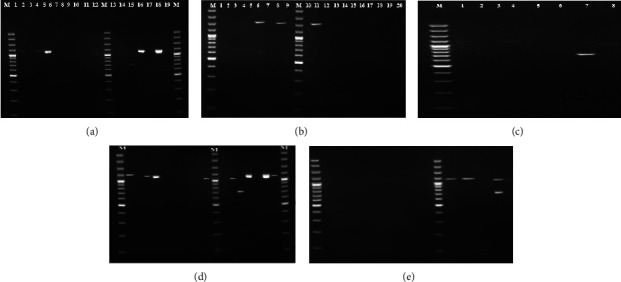
(a) Ladder size: 3000 bp, lines 1–3–4–5–12–14–16–18–19: *cnf1* positive (1111 bp); line 15: *cnf3* positive (757 bp). (b) Ladder size: 3000 bp, lines 6–8–11 *cnf2* positive (1240 bp). (c) Ladder size: 3000 bp, line 4, 5, 6, and 8 *cnf1* positive (1111 bp), line 1; both *cnf1* positive (1111 bp) and *cnf2* positive (1240 bp), line 7; both *cnf1* positive (1111 bp) and *cnf3* positive (757 bp). (d) Ladder size: 3000 bp, line 1, 3, 4, 9, 11, 13, 15, and 16, *cnf1* positive (1111 bp), line 12, *cnf3* positive (757 bp). (e) Ladder size: 3000 bp, line 18, both *cnf1* positive (1111 bp) and *cnf3* positive (757 bp).

**Figure 4:**
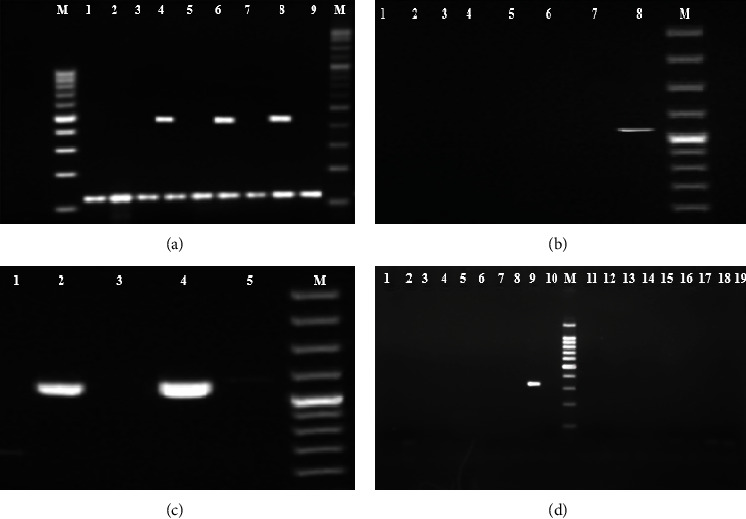
(a) Ladder size: 1000 bp, line 4, 6, and 8 *cdt1* positive (411 bp); lines 1–9 *uidA* positive (102 bp). (b) Ladder size: 1000 bp, line 8 *cdt2* (556 bp). (c) Ladder size: 1000 bp, line 2, 4, and 5 *cdt3* (555 bp). (d) Ladder size: 1000 bp, line 9, *cdt4* positive (350 bp).

**Figure 5:**
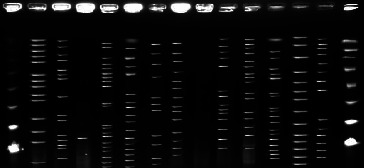
PFGE resulting band profiles.

**Figure 6:**
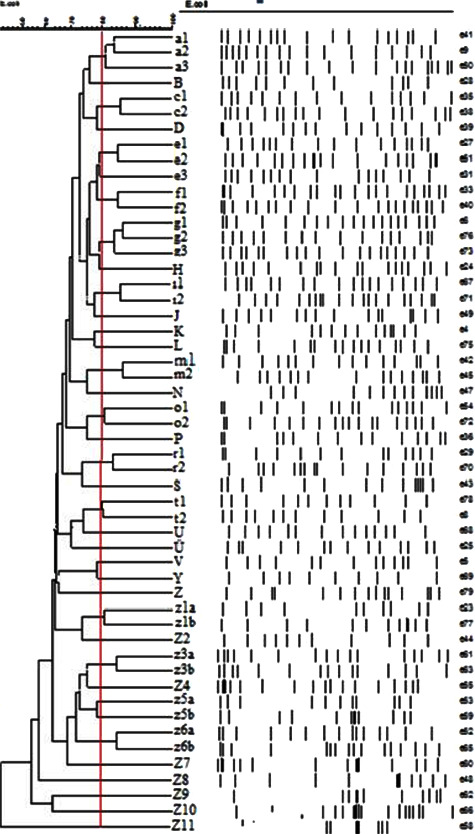
Simple dendogram of PFGE.

At least one virulence gene was detected in 156 (24%) of the 646 ExPEC isolates. We detected cnf1, cnf2, and cnf3, in 78, 12, and 20 ExPEC isolates, respectively (Figures 3(a)–3(e)). cdt1, cdt2, cdt3, and cdt4 genes were present in 20, 4, 4, and 4 of the isolates (Figures 4(a)–4(d)), and at least two factors cnf1-cnf3, cnf1-cdt1, and cdt1–cdt4 existed in 6, 4, and 4 of the isolates, respectively (Table 3). These 156 isolates were distributed in 106 large clusters by PFGE (Figures 5 and 6). Virulent ExPEC is primarily related to groups *B*2 (60%) and *D* (32%).

One hundred sixteen (18%) of these isolates harbored one of the genes encoding *CNF*: *cnf1* (*n* = 78), *cnf2* (*n* = 12), *cnf3* (*n* = 20), and *cnf1* + *cnf3* (*n* = 6) (Figures 3(c) and 3(e)). However, 36 (5.4%) isolates had *CDT* only: *cdt1* (*n* = 20), *cdt2* (*n* = 4), *cdt3* (*n* = 4), *cdt4* (*n* = 4), and *cdt1* + *cdt4* (*n* = 4) (Figures 4(a)–4(d)). Interestingly, four (0.6%) had both toxin factors.

According to the clinical origins of 646 ExPEC isolates, the *CDT* gene family was found to be 3.6% in the urinary tract samples and 13.7% in the nonurinary isolates. The *CNF* gene family was at an 18% rate in the urinary tract samples (Table 3).

In 156 ExPEC isolates in which at least one virulence factor had been detected, the most frequently isolated gene was *cnf1 *, with a rate of 50% + 6.3% in binary combinations, followed by *cdt1,* with a rate of 12.8% + 5% in binary combinations (Table 4).

The distribution of *CNF* and *CDT* genes of 156 ExPEC isolates according to the origin of the isolate as urinary and nonurinary samples is presented in Table 5. Of the 156 ExPEC isolates, 121 (78%) were of urinary tract origin, while 35 (22%) were extraurinary. The most common virulence genotypes in 121 urinary tract specimens were those of the *CNF* gene family, with a rate of 84.3%. In the urinary samples, the range of the *CDT* gene family was 18.3%. The positivity rate of both *CNF* and *CDT* genes at the same time in urine samples was only 2.4% (Table 5). While 68 (87.2%) of 78 *cnf1*-positive isolates originated from the urinary tract, 10 (12.8%) of them were isolated from other extraintestinal sites. On the other hand, among the *CDT* genes (*cdt1, cdt2, cdt3,* and *cdt4*), the most common isolate in urine samples was *cdt1*, with a rate of 10% (12/121) (Table 5).

The phylogenetic analyses of 156 ExPEC isolates revealed that these isolates consisted of four main phylogenetic groups, including *A*, *B*1, *B*2, and *D*. In 156 ExPEC isolates, the most frequently analysed phylogenetic group was *B*2 (60.8%), followed by *D* (30.7%), *A* (5.12%), and *B*1 (3.2%) (Table 6).

Of 68 *cnf1* isolates originating from the urinary system, while 38 were in the *B*2 and 30 were in the *D* phylogenetic group. No *cnf1* isolate was found in the A phylogenetic group. Only three isolates of *cnf1* originating from the nonurinary sites were in the *B*1 phylogenetic group (Table 6).

**Figure 7:**
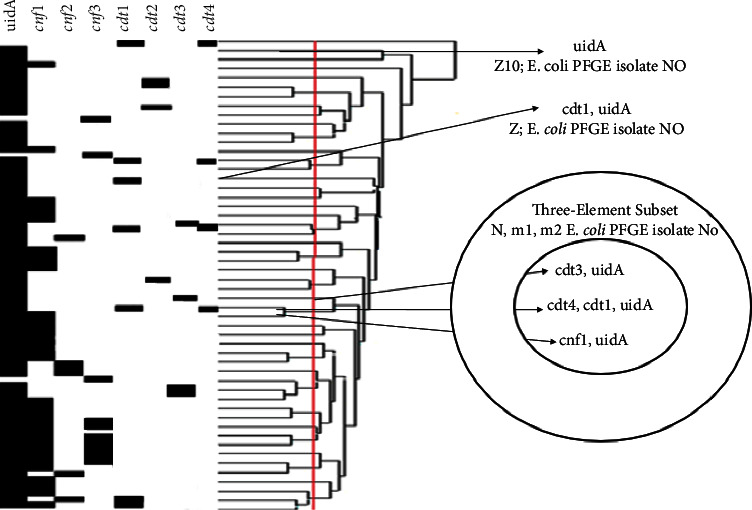
Clustering of virulence genotypes.

To determine the phylogenetic relationships of 156 ExPEC isolates, whole-genome DNA fragment polymorphism analysis was performed with XbaI-PFGE (Figure 5). According to this method, based on 80% similarity, 156 ExPEC isolates were collected in 106 clusters. Four of the test isolates (*Z*23, *Z*25, *Z*36, and *Z*38) had five members; eight of them (*A*, *E*, *G*, *Z*60, *Z*64, *Z*80, *Z*83, and *Z*88) had three members; and 15 of them had two members (*C*, *F*, *I*, *M*, *O*, *R*, *Z*2, *Z*3, *Z*5, *Z*6, *Z*24, *Z*56, *Z*58, *Z*73, and *Z*76) (Figure 6). In addition, 82 isolates were unrelated, and each of them was present as a “singleton” (Figure 6). The isolates accumulated in the single-member cluster and the same subcluster had different virulence factors. Two specimens were obtained from one patient with a diagnosis of urosepsis; one was blood, and the other was urine. These two specimens were distributed in different clusters and had different virulence factors. While the sample sourced from blood was positive in terms of *cdt1* virulence factor and showed *Z* polymorphism, the other sourced from urine was negative in terms of any virulence factor and showed *Z*10 polymorphism, using the PFGE method. The clustering of the virulence genotype is shown in “Figure 7” as a simple representative dendrogram.

The data that support the findings of this study are available on request from the corresponding author (Cansu Onlen Guneri). The data are not publicly available due to participant privacy.

## 4. Discussion

To our knowledge, this is the first study that showed *cnf2, cnf3, cdt1, cdt2, cdt3*, and *cdt4* genes in Turkey. We used *CNF-* and *CDT*-specific primers to determine the prevalence of *CNF*-*CDT*s, which were proven to increase the colonization of ExPEC isolates outside the intestinal tract. There are a limited number of clinical-epidemiological studies in the literature that suggest *CNF*-*CDT* causes changes in host cell functions, damage to epithelial cells, and potent virulence factors that may lead to tissue pathologies, such as stimulating the inflammatory response [9].

While the rate of *CDT* found in this study was consistent with some previously reported studies [8, 9], there were also studies reporting low rates, such as 0.9–2.5% [12, 13]. The different *CDT* gene ratios between our study and others may be explained by the difference in the origins of the study materials (Table 3). In this study, the *CDT* genes were higher in nonurinary tract specimens compared with urinary tract samples. Also, approximately 80% of the samples included in our study were urine. It could be explained by the high prevalence of *E. coli* isolates in urinary tract infections by the easy colonization of the intestinal isolates by invasion into the urinary system through the neighbourhood relationship. It could also be explained by the fact that they can cause disease in the host under different stress factors because they can easily colonize without the need for an extra virulence factor.

Besides the *CDT* gene, we also investigated all *CDT* alleles (*cdt1, cdt2, cdt3*, and *cdt4*). We found that the frequency of *cdt1* and *cdt4* was higher than that of *cdt2* and *cdt3* in all ExPEC isolates (Table 4). Previously published studies also reported that the *cdt1* and *cdt4* alleles were positive, while the *cdt2* and *cdt3* alleles were negative [8, 12–15]. These results suggest that the *cdt1* and *cdt4* alleles of the *CDT* genes show very common and close homology and may have been derived from a common ancestor by phage transduction.

On the other hand, the rates of *CNF* genes and alleles that we detected in the present study could be similar to some studies; their rates were between 5–34% [16, 17], and such a wide range of outcomes may be attributed to unreported patient characteristics.

One of the most important results of this study was the high levels of *CNF* genes, especially in urine samples. The results of the present study were higher than in most other studies. In other studies, these rates have changed between 16.5% and 61.9% [9, 12, 13, 18, 19]. In a study conducted in Turkey by Bozcal et al., the *cnf1* ratio was 12% in ExPEC isolates obtained from blood samples [20]. When only considering blood samples in our study, this rate was 11% (3/28). We can say that our results were consistent with Bozcal's study. These results suggest that *cnf1* can be a specific marker for virulent isolates, especially for the urinary tract and sepsis-related ExPEC.

In the present study, *cnf1* was the most common virulence factor compared to *cnf2* and *cnf3*. We can explain this by that the fact that *cnf2* and *cnf3* are of animal origin and rare in humans. However, in our study, the rates of *cnf2* and *cnf3* were higher than in previous studies [9, 16, 17, 19, 21].

The phylogenetic distributions of ExPEC isolates in our study were similar to previous studies. According to these results, we can say that ExPEC originated predominantly from phylogroup *B*2 and, to a lesser extent from, group *D* [9, 10, 12, 16, 17, 22]. However, in the study conducted by Bozcal et al., in ExPEC isolates obtained from blood samples, these rates were *D* (38.14%), *A* (29.89%), *B*2 (20.61%), and *B*1 (11.34%) [6]. The results of our work are inconsistent with the Bozcal study. It may depend on the fact that the samples in our study were from different sites and the high number of specimens studied.

Finally, to understand the *CNF*-*CDT* positive *E. coli* isolates that evolved from deletion, recombination mutations, or evolved from an ancestor strain and acquired the ExPEC feature, we used PFGE in these ExPEC isolates. The results of PFGE in the present study regarding similarity rates and clusters have shown differences from studies conducted by Oloomi and Bouzari [23] and Mora et al. [24]. In our study, in the aspect of the XbaI-PFGE results, the phylogenetic relationships of ExPEC isolates were found to be very weak. ExPEC isolates with the same phylogenetic properties may have different virulence characteristics (Figure 7). As shown in the results section of our study, two ExPEC isolates obtained from one patient from different clinical samples at the same time may have originated from different ancestors evolutionarily. We could explain this situation by saying that most extraintestinal *E. coli* infections have been acquired from the community. The characteristics and spread of virulent ExPEC isolates should be monitored by molecular surveillance and limited by vaccine studies.

## 5. Limitations

There were some limitations to the present study. The first limitation was the lack of detailed data on clinical features, although the diagnosis of the patients was presented. Thus, we could not compare the clinical course and antibiotic susceptibility of the patients with positive virulence genes to negative ones. The second limitation was that the virulence genes we detected in extraintestinal *E. coli* isolates could also be investigated in intestinal isolates. The third limitation was that we could not show antitoxin in susceptible cell types like Vero by cell culture studies. Finally, standard controls could not be obtained due to the limited number of similar studies in our country. Despite efforts for international cooperation, we could not have the standard isolates.

## 6. Conclusion

To our knowledge, we showed cnf2, cnf3, cdt1, cdt2, cdt3, and cdt4 genes for the first time in Turkey. The widespread presence of the CNF gene in *E. coli* can help distinguish ExPEC from commensal isolates. These genes may also be a specific marker for noncommensal *E. coli*. In particular, the presence of cnf1 increases the *E. coli* colonization in the urinary system and could be a characteristic virulence factor for the uropathogenic *E. coli* isolates. Virulence factors can be distributed depending on phylogenetic groups (*A*, *B*1, *B*2, and *D*), and these phylogroups may be used to distinguish between pathogen and nonpathogen isolates.

## Figures and Tables

**Table 1:** Oligonucleotide primers are used in this study.

Primer	Oligonucleotide sequence (5′–3′)	Specificity	PCR product size (bp)	References
*uidA* F *uidA* R	ATGCCAGTCCAGCGTTTTTGC AAAGTGTGGGTCAATAATCAGGAAGTG	*uidA*	102	Abid and Al-Zuwainy [7]
*cnf1* F *cnf1* R	GGGGGAAAGTACAGAAGAATTA TTGCCGTCCACTCTCACCAGT	*cnf1*	1111	Dubois et al., [8]
*cnf2* F *cnf2* R	TATCATACGGCAGGAGGAAGCACC GTCACAATAGACAATAATTTTCCG	*cnf2*	1240	
*cnf3* F *cnf3* R	TAACGTAATTAGCAAAGA GTCTTCATTACTTACAGT	*cnf3*	757	
*cdt1* F *cdt1* R	CAATAGTCGCCCACAGGA ATAATCAAGAACACCACCAC	*cdt1*	411	Toth et al., [9]
*cdt2* F *cdt2* R	GAAAGTAAATGGAATATAAATGTCCG TTTGTGTTGCCGCCGCTGGTGAAA	*cdt2*	556	
*cdt3* F *cdt3* R	GAAAGTAAATGGAATATAAATGTCCG TTTGTGTCGGTGCAGCAGGGAAAA	*cdt3*	555	
*cdt4* F *cdt4* R	CCTGATGGTTCAGGAGGCTGGTTC TTGCTCCAGAATCTATACCT	*cdt4*	350	
*chuA* F *chuA* R	GACGAACCA ACGGTCAGGAT TGCCGCCAGTACC AAAGACA	*chuA*	279	Clermont et al., [10]
*yjaA* F *yjaA* R	TGAAGTGTCAGGAGACGCTG ATGGAGAATGCGTTCCTCAAC	*yjaA*	211	
*TspE4C2* F *TspE4C2* R	GAGTAATGTCGGGGCATTCA CGCGCCAACAAAGTATTACG	*TspE4C2*	152	

**Table 2:** Thermal cycles for eleven primers are used in this study.

No	Step	uidA	CNFs	CDTS	chuA, yjA, tspe4.c2
1	Initial denaturation	94°C/5 min	94°C/3 min	94°C/5 min	94°C/5 min
2	Denaturation	94°C/30 sec	94°C/1 min	94°C/1 min	94°C/30 sec
	Annealing	63°C/30 sec	60°C/1 min	60°C/1 min	60°C/30 sec
	Extension	72°C/1.5 min	72°C/1 min	72°C/1 min	72°C/1.5 min
3	Final extension	72°C/5 min	72°C/7 min	72°C/10 min	72°C/5 min

**Table 3:** The distribution of toxin genes in 646 ExPEC isolates.

VFs (%)	All isolates (*n* = 646) *n* (%)	Urinary system *n* = 522 (80)	Nonurinary system						
			Total *n* = 124 (20)	Wound (*n* = 54)	Blood (*n* = 28)	Sputum (*n* = 21)	Aspirate (*n* = 6)	Sterile body fluid (*n* = 12)	Vagen (*n* = 3)
*cnf1*	78 (12)	68 (13)	10 (8)	3 (5)	3 (11)	3 (14)	1 (16)	—	—
*cnf2*	12 (2)	10 (2)	2 (1, 6)	1 (2)	1 (4)	—	—	—	—
*cnf3*	20 (3)	16 (3)	4 (2, 3)	2 (4)	1 (4)	1 (5)	—	—	—
*cnf1*–*cnf3*	6 (0, 9)	5 (1)	1 (0,8)	—	1 (4)	—	—	—	—
Total *cnfs*	116 (18)	99 (20)	17(13)						
*cdt1*	20 (3)	12 (2)	8 (7)	—	2 (8)	2 (10)	2 (31)	2 (16)	—
*cdt2*	4 (0,6)	2 (0,3)	2 (1, 7)	1 (2)	1 (4)	—	—	—	—
*cdt3*	4 (0,6)	2 (0,3)	2 (1, 7)	—	—	1 (5)	—	—	—
*cdt4*	4 (0,6)	2 (0,3)	2 (1, 7)	1 (2)	—	—	1 (16)	—	—
*cdt1*–*cdt4*	4 (0,6)	1 (0,2)	3 (2, 3)	2 (4)	1 (4)	—	1 (16)	—	—
Total *cdts*	36 (4, 5)	19 (3, 6)	17 (13.7)						
*cnf1-cdt1*	4 (0,9)	3 (0,8)	1 (1, 7)	1 (2)	—	—	—	—	—

**Table 4:** The distribution of toxin genes in *CDT-* and *CNF*-positive 156 ExPEC isolates.

Virulence factor	*cnf1*	*cnf2*	*cnf3*	*cnf1-cnf3*	*cdt1*	*cdt2*	*cdt3*	*cdt4*	*cdt1-cdt4*	*cnf1-cdt1*
Total (*n* = 156)	78	12	20	6	20	4	4	4	4	4
*n*/total	78/156	12/156	20/156	6/156	20/156	4/156	4/156	4/156	4/156	4/156
%	50	7.6	12.8	3.8	12.8	2.5	2.5	2.5	2.5	2.5

**Table 5:** The distribution of factor-toxin genes of 156 ExPEC isolates by clinical origin/the virulence factor distributions in the urinary and nonurinary tract foci in *CDT*- and *CNF*-positive ExPEC isolates.

*N* (%) urinary system *n* = 121 (78)		*N* (%) nonurinary system *n* = 35 (22)					
Isolates VFs	Urine (*n* = 121)	Wound (*n* = 11)	Blood (*n* = 9)	Sputum (*n* = 7)	Aspirate (*n* = 5)	CSF (*n* = 1)	Peritoneal fluid (*n* = 1)
*cnf1*	68 (56)	3 (27)	3 (32)	3 (43)	1 (20)	—	—
*cnf2*	10 (8)	1 (9)	1 (11)	—	—	—	—
*cnf3*	16 (13)	2 (18)	1 (11)	1 (14)	—	—	—
*cnf1*–*cnf3*	5 (4)	—	1 (11)	—	—	—	—
*cdt1*	12 (10)	—	2 (22)	2 (29)	2 (40)	1	1
*cdt2*	2 (2)	1 (9)	1 (11)	—	—	—	—
*cdt3*	2 (2)	—	—	1 (14)	1 (20)	—	—
*cdt4*	2 (2)	1 (9)	—	—	1 (20)	—	—
*cdt1*–*cdt4*	1 (1)	2 (18)	1 (11)	—	—	—	—
*cnf1-cdt1*	3 (2)	1 (9)	—	—	—	—	—

**Table 6:** The distribution of 156 ExPEC isolates virulence genes by phylogenetic group and clinical origin.

Virulence genes	No. (%) of virulence isolates (*n* = 156)		No. (%) of isolates							
	Urinary/others (*n* = 121)/(*n* = 35)		Group *A n* = 8 (5, 1)		Group *B*1 *n* = 5 (3, 2)		Group *B*2 *n* = 95 (60)		Group *D n* = 48 (30)	
			Urinary/others		Urinary/others		Urinary/others		Urinary/others	
*cnf1*	68 (56)	10 (29)	0	0	0	3 (60)	38 (39)	3 (4)	30 (62)	4 (8)
*cnf2*	10 (8)	2 (6)	3 (37)	0	0	0	7 (7)	2 (2)	0	0
*cnf3*	16 (13)	4 (11)	2 (25)	2 (25)	0	0	12 (13)	2 (2)	2 (4)	0
*cdt1*	12 (10)	8 (23)	0	1 (13)	0	0	12 (13)	7 (7)	0	0
*cdt2*	2 (2)	2 (6)	0	0	0	0	0	0	2 (4)	2 (4)
*cdt3*	2 (2)	2 (6)	0	0	0	0	0	0	2 (4)	2 (4)
*cdt4*	2 (2)	2 (6)	0	0	0	2 (40)	2 (2)	0	0	0
*cnf1*–*cnf3*	5 (4)	1 (3)	0	0	0	0	5 (5)	1	0	0
*cdt1*–*cdt4*	1 (1)	3 (9)	0	0	0	0	1 (5)	3 (3)	0	0
*cnf1-cdt1*	2 (2)	2 (6)	0	0	0	0	0	0	2 (4)	2 (4)

## Data Availability

The data used to support the findings of this study are available from the corresponding author upon reasonable request.
